# Continuous-cropping-tolerant soybean cultivars alleviate continuous cropping obstacles by improving structure and function of rhizosphere microorganisms

**DOI:** 10.3389/fmicb.2022.1048747

**Published:** 2023-01-04

**Authors:** Wenbo Liu, Nan Wang, Xingdong Yao, Dexin He, Hexiang Sun, Xue Ao, Haiying Wang, Huijun Zhang, Steven St. Martin, Futi Xie, Jingkuan Wang

**Affiliations:** ^1^Soybean Research Institute, Shenyang Agricultural University, Shenyang, China; ^2^Postdoctoral Station of Agricultural Resources and Environment, Land and Environment College, Shenyang Agricultural University, Shenyang, China; ^3^Key Laboratory of Soybean Biology of Chinese Education Ministry, Soybean Research Institute, Northeast Agricultural University, Harbin, China; ^4^Department of Horticulture and Crop Science, The Ohio State University, Columbus, OH, United States

**Keywords:** soybean, continuous cropping, soil nutrient content, enzyme activity, soil bacterial community, soil bacterial function

## Abstract

**Introduction:**

Soybean continuous cropping will change soil microorganisms and cause continuous cropping obstacles, resulting in a significant yield decline. Different soybean cultivars have different tolerances to continuous cropping, but the relationship between continuous cropping tolerance and soil microorganisms is not clear.

**Methods:**

Two soybean cultivars with different tolerances to continuous cropping were used to study the effects of continuous cropping on soil physical and chemical properties, nitrogen and phosphorus cyclic enzyme activities, rhizosphere soil microbial community and function.

**Results:**

The results showed that the yield reduction rate of a continuous-cropping-tolerant cultivar (L14) was lower than that of a continuous-cropping-sensitive cultivar (L10) under continuous cropping. At R1 and R6 growth stages, soil nutrient content (NH_4_^+^-N, NO_3_^−^-N, AP, DOM, TK, and pH), nitrogen cycling enzyme (URE, NAG, LAP) activities, phosphorus cycling enzyme (ALP, NPA, ACP) activities, copy numbers of nitrogen functional genes (*AOA*, *AOB*, *nirK*, *nirK*) and phosphorus functional genes (*phoA*, *phoB*) in L14 were higher than those in L10. Soybean cultivar was an important factor affecting the structure and functional structure of bacterial community under continuous cropping. The relative abundances of *Proteobacteria*, *Bacteroidota*, *Acidobacteriota* and *Verrucomicrobiota* with L14 were significantly higher than those of L10. The complexity of the soil bacterial community co-occurrence network in L14 was higher than that in L10.

**Discussion:**

The continuous-cropping-tolerant soybean cultivar recruited more beneficial bacteria, changed the structure and function of microbial community, improved soil nitrogen and phosphorus cycling, and reduced the impact of continuous cropping obstacles on grain yield.

## 1. Introduction

In recent years, continuous cropping has become the most common practice for soybean production in China. Especially in northeast China, the phenomenon of continuous cropping is widespread due to the limited cultivated land area. Continuous cropping will lead to the change of soil physical and chemical properties, the increase of pathogenic bacteria, the decrease of soil enzyme activities and the destruction of soil microbial community (Seifert et al., 2017), which is further manifested as a continuous cropping obstacle (Wu et al., 2015).

Soil microorganisms are an important part of terrestrial ecosystems (Schloss and Handelsman, 2006). The soil bacterial community plays a key role in promoting organic matter decomposition, nutrient cycling, inhibiting soil-borne diseases and promoting plant growth (Bardgett and van der Putten, 2014), which is beneficial to maintaining soil quality, agricultural sustainability and ecosystem multifunctionality. The composition and function of soil bacterial communities influence soil properties. Proper management of agricultural fields enables soil microorganisms to better perform their diversified ecological functions (Amorim et al., 2020; Kelly et al., 2020). Cropping system is the main factor that changes microbial community structure (Meriles et al., 2009; Zhou et al., 2018; Liu et al., 2019). The response of microbial species to soybean cropping system was different in different studies. In one case, continuous cropping reduced the total amount of bacteria and actinomycetes and increased the number of fungi (Yu et al., 2014). In another study, the bacterial community in soybean rhizosphere soil changed little (Li et al., 2010). In several experiments the abundance and diversity of microorganisms were reduced under continuous cropping (Wardle et al., 2004; Tang et al., 2009; Zhu et al., 2010). Soil sterilization significantly improved soybean plant growth compared with unsterilized continuous cropping soil, proving that soil microorganisms were the main cause of soybean continuous cropping obstacles (Dias et al., 2015).

There were significant differences in soil microorganisms among different genotypes of the same crop. Research showed that different genotypes of crop had significant differences in soil microorganisms (Luo et al., 2005; Zhao et al., 2006; Ma et al., 2007; Zhu et al., 2010). It has also been reported that rhizosphere microorganisms differed significantly among different soybean genotypes (Qian et al., 2010), which affected crop productivity, soil nutrient cycling (Schmidt et al., 2019; Priyanka et al., 2020; Benitez et al., 2021) and plant disease resistance (Thaddeus et al., 2017). There were interaction effects between crops with different genotypes and soil microorganisms, which affected the productivity of crops with different genotypes. Under continuous cropping, the yield reduction rate of different soybean cultivars was different, which indicated that different cultivars had different tolerance to continuous cropping. However, the relationship between the tolerance of soybean cultivars and soil microorganisms has not been reported. Therefore, this study on the effect of continuous cropping on rhizosphere microbial community of soybean cultivars with different tolerances to continuous cropping can provide theoretical basis for field management of continuous cropping soybean.

In this study, two soybean cultivars with different tolerances to continuous cropping were used, and three treatments of crop rotation (CR), continuous cropping (CC) and continuous cropping + compound fertilizer (CF) were applied, the soil bacterial community and functional structures of soybean cultivars with different continuous cropping tolerance were compared by high-throughput sequencing technology, and the relationship between soybean cultivars with different continuous cropping tolerance and soil microorganisms was discussed.

## 2. Materials and methods

### 2.1. Experimental design

The experiment was conducted in 2021 at the Liaozhong Positioning Test Base (41°52’N, 122°72’W, elevation 5.5–23.5 m) of Shenyang Agricultural University, Liaoning Province. Before this experiment, the field was used under soybean continuous cropping and corn-corn-soybean rotation for 5 years. The climate in this site belongs to subhumid continental climate in south temperate zone, with the mean annual precipitation of 640 mm, mean annual temperature of 8°C and mean annual sunshine is 2,527 h. The basic fertility of soil was 1.26 g kg^−1^ of total nitrogen (TN), 11.15 g kg^−1^ of organic matter (DOM), 37.30 mg kg^−1^ of available phosphorus (AP) and 161.78 mg kg^−1^ of available potassium (AK). Our previous screening experiments showed that Liaodou 14 was a continuous-cropping-tolerant soybean cultivar and Liaodou 10 was a continuous-cropping-sensitive soybean cultivar.

The experiment was a two-factor randomized block design, and the two factors were different cropping systems treatments [crop rotation (no fertilizer), continuous cropping (no fertilizer), continuous cropping + compound fertilizer (pure nitrogen: 90 kg ha^−1^; pure phosphorus: 90 kg ha^−1^; pure potassium: 90 kg ha^−1^)] and different soybean cultivars [continuous-cropping-tolerant soybean cultivar Liaodou 14 (L14) and continuous-cropping-sensitive soybean cultivar Liaodou 10 (L10)] with 3 replicates, 18 plots with 5 rows per plot, and the soybean seedling density was 150,000 plants ha^−1^. Seeds were selected before sowing, 4 seeds per hole, sowing depth 3 cm, thinning at seedling stage, leaving 2 plants per hole, fertilizer was applied once at sowing time, as in conventional field management.

### 2.2. Field sampling of soil

At the beginning bloom growth stage (R1) and the full seed growth stage (R6), five spots were selected in the S-shaped manner, and the soil attached to the roots of soybean plants (i. e. rhizosphere soil) was brushed with a sterile brush and mixed fully to form a composite sample, which was stored at −80°C and used for sequencing of soil microorganisms.

In each plot, 5 spots (5 cm diameter × 20 cm depth) were taken in an S-shape and mixed thoroughly to form a composite sample. The soil sample was sifted through a 2 mm sieve to remove crop residues such as leaves and roots. Each sample was then divided into two parts: One part was stored at −80°C for the determination of functional genes for nitrogen and phosphorus cycling enzymes and soil enzyme activities, and the other part was used for the soil property analysis.

### 2.3. Test methods

#### 2.3.1. Determination of soil physical and chemical indexes

Soil water content (WC) in 0–20 cm soil layer was determined by drying method. The soil pH was measured by pH-meter method, and the soil-water mass volume ratio was 1:5. Nitrate (NO_3_^−^-N) was determined by UV spectrophotometry method, ammonium (NH_4_^+^-N) was determined by indophenol blue method, AP was determined by flame atomic absorption spectrophotometer method, and DOM was determined by dichromate oxidation and ferrous sulfate titrimetric method (Jangid et al., 2011; McDaniel and Grandy, 2016). TN was determined by automated Kjeldahl apparatus (KJELTECTM8400). The total phosphorus (TP) was determined by molybdenum-antimony anti-colorimetric method. The total potassium (TK) was determined by flame atomic absorption spectrophotometer method.

#### 2.3.2. Determination of soil enzyme activities

Soil urease (URE) activity was measured by indophenol blue method (Liao and Raines, 1985). Soil N-acetyl-β-glucosaminidase (NAG), leucine aminopeptidase (LAP), alkaline phosphatase (ALP), neutral phosphatase (NPA) and acid phosphatase (ACP) activities were measured by microplate fluorescence detection technology (Saiya-Cork et al., 2002).

#### 2.3.3. Extraction of soil total DNA

Genomic DNA was extracted from the samples by CTAB or SDS according to the manufacturer’s recommendations, and then the purity and concentration of DNA was determined by agarose gel electrophoresis, and the sample DNA was diluted to 1 ng μL^−1^ using sterile water.

#### 2.3.4. Quantitative fluorescence qPCR assay

The Platinum® Taq DNA Polymerase (Thermo Fisher) enzyme and specific primers were used to quantify the target genes. Standard curves were constructed by 10-fold dilution of plasmids containing target genes, and real-time fluorescence quantification was performed on a PCR instrument (7,500, ABI, United States). A 15 μl reaction system was used for RT-qPCR analysis (Zhou et al., 2019), including 3 μl 100 ng DNA, 1.2 μl 10 μM PCR forward primer, 1.2 μl 10 μM reverse primer, 7.5 μl SYBR qPCR mix and 2.1 μl ddH_2_O. Primer sequences are shown in Supplementary Table S1.

#### 2.3.5. PCR amplification and 16S rDNA sequencing

Using diluted genomic DNA as template, specific primers with Barcode, Phusion® High-Fidelity PCR Master Mix with GC Buffer from New England Biolabs company, were used according to the selection of sequencing region, and high efficient, high fidelity enzyme for PCR, to ensure amplification efficiency and accuracy. 515F (5’-CCTAYGGGRBGCASCAG-3′) and 806R (5’-GGACTACNNGGGTATCTAAT-3′) were selected as sequencing primers to amplify the V4 region of 16S rRNA gene (Walters et al., 2016). The PCR products were detected by 2% agarose gel electrophoresis, purified by magnetic beads, and quantified by enzyme-labeled. The PCR products were mixed in equal amounts according to the concentration of PCR products, and the fully mixed PCR products were detected by 2% agarose gel electrophoresis again. The target band was recovered by using the gel recovery kit provided by qiagen. TruSeq® DNA PCR-Free Sample Preparation Kit was used for library construction. The constructed library was quantified by Qubit and Q-PCR, and the qualified libraries were sequenced using the Illumina NovaSeq sequencing platform at MetWare.^1^

### 2.4. Data analysis

The sample data to Barcode and primer sequences, using FLASH (V1.2.7,^2^) (Magoč and Steven, 2011) reads from samples of each for strict filtering processing (Bokulich et al., 2013) get high quality data Tags. Reference Qiime (V1.9.1,^3^) (Caporaso et al., 2010) Tags quality control process, remove the processing of chimeric sequences, Tags sequence through annotations^4^ (Rognes et al., 2016) and species database for matching detection chimeric sequences, get the Effective Tags. The Effective Tags of all samples were clustered into OTUs (Operational Taxonomic Units) with 97% Identity, and the sequence with the highest frequency was screened as the representative sequence of OTUs. The least amount of data was used as the standard for homogenization. Alpha Diversity was used to analyze microbial community diversity in samples (Li et al., 2013), and Qiime software (Version 1.9.1) was used to calculate Observed_species, Shannon, Simpson, Chao1 and ACE indices. Beta Diversity was used to compare and analyze the microbial community composition of different samples, and the differences among different samples were found by Principal Co-ordinate Analysis (PCoA). FAPROTAX was a prokaryotic environmental function database for the analysis of bacterial function. Based on species abundance, Spearman correlation coefficient (SCC) between each genus of bacteria was calculated, and the correlation coefficient matrix was obtained to filter out the connection node self-connection with cutoff value (<0.6) weakly correlated and the connection node abundance (<0.005%) (Barberán et al., 2012). According to the filtered correlation values, with bacteria as nodes and values as edges, graphviz-2.38.0 was used to draw the network diagram (Bastian et al., 2009).

Data were processed by Excel 2010, and the difference in significance was analyzed by using the statistical software SPSS version 22.0 (SPSS Inc., Chicago, IL, United States). The redundancy analysis (RDA) was performed using CANOCO software 4.5. LDA Effect Size (LEfSe) software was used for LEfSe, and the default screening value of LDA Score is set to 4.

## 3. Results

### 3.1. Effects of continuous cropping on soil nutrients and yield of soybean cultivars with different tolerances

Yield and biomass were significantly different among cultivars and cropping systems, but there was no significant cultivars × cropping systems interaction. Continuous cropping induced significantly decreased soybean yield and biomass, and fertilization increased yield and biomass under continuous cropping. In addition, L14 had higher yield (27.17%), biomass (19.42%), and lower yield reduction rates (13.15%) than L10 under CC treatment (Table 1).

**Table 1:** Effects of crop rotation and continuous cropping on soybean productivity.

Yield and biomass	Cultivar	CR	CC	CF	Mean	Cultivar	Cropping system	Cultivar × Cropping system
Yield (kg ha^−1^)	L10	2,636	916	1,638	1730(864)b	**	**	ns
	L14	3,130	1,328	2,145	2,201(902)a			
	Mean	2,883(349)a	1,124(292)c	1891(359)b				
Biomass (kg ha^−1^)	L10	11,521	5,954	7,380	8,285(2892)b	*	**	ns
	L14	12,952	6,803	9,926	9,894(3074)a			
	Mean	12,237(1102)a	6,379(600)c	8,653(1800)b				

Different lowercase letters indicate significant difference among treatments (*p* < 0.05). *p < 0.05; **p < 0.01.

At the R1 growth stage, NH_4_^+^-N, NO_3_^−^-N, AP, DOM, TK, TP, WC content, and pH value in soil were significantly different among cultivars and cropping systems, and NO_3_^−^-N, AP, WC content and pH value showed significant cultivars × cropping systems interactions. Continuous cropping induced significantly increased pH value and significantly decreased NH_4_^+^-N, NO_3_^−^-N, DOM, TK, TP, TN, and WC content. Fertilization increased NH_4_^+^-N, NO_3_^−^-N, AP, DOM, TK, TP, and TN content in soybean soil under continuous cropping. L14 had significantly higher contents of NH_4_^+^-N, NO_3_^−^-N, AP, DOM, TK, TP, TN, WC, and pH value than L10 under CC treatment (Supplementary Table S2).

At the R6 growth stage, NH_4_^+^-N, NO_3_^−^-N, AP, DOM, TK content, and pH value in soil were significantly different among cultivars and cropping systems, while TK and WC content displayed cultivars×cropping systems interactions. Continuous cropping induced significantly increased pH value and WC content, and significantly decreased NH_4_^+^-N, NO_3_^−^-N, DOM, TK and TN content. Fertilization increased NH_4_^+^-N, NO_3_^−^-N, AP, DOM, TK, TP, TN and WC content in soybean soil under continuous cropping. L14 had significantly higher contents of NH_4_^+^-N, NO_3_^−^-N, AP, DOM, TK and pH value than L10 under CC treatment (Supplementary Table S3).

### 3.2. Effects of continuous cropping on soil enzyme activities of soybean cultivars with different tolerances

At the R1 growth stage, URE, NAG, LAP, ALP, NPA, and ACP activities were significantly different among cultivars and cropping systems, and URE activity was showed a significant cultivars × cropping systems interaction. Continuous cropping induced significantly decreased NAG, LAP, ALP, NPA, and ACP activities, and fertilization increased URE, NAG, LAP, ALP, NPA, and ACP activities under continuous cropping. L14 showed significantly higher URE, NAG, LAP, ALP, NPA, and ACP activities than L10 under CC treatment (Table 2).

**Table 2:** Effects of crop rotation and continuous cropping on soil enzyme activity parameters.

Soil enzyme activity	Cultivar	CR	CC	CF	Mean	Cultivar	Cropping system	Cultivar × Cropping system
R1 growth stage								
URE (mg d^−1^ g^−1^)	L10	1.12	1.16	1.40	1.23 (0.16)b	**	**	*
	L14	1.42	1.31	1.73	1.48 (0.20)a			
	Mean	1.27 (0.17)b	1.23 (0.14)b	1.56 (0.18)a				
NAG (nM h^−1^ g^−1^)	L10	14.81	10.14	14.15	13.03 (2.31)b	**	**	ns
	L14	17.13	12.13	15.12	14.79 (2.35)a			
	Mean	15.97 (1.50)a	11.13 (1.37)c	14.63 (1.04)b				
LAP (nM h^−1^ g^−1^)	L10	21.03	18.08	15.95	18.35 (2.37)b	**	**	ns
	L14	24.07	20.00	18.50	20.86 (2.81)a			
	Mean	22.55 (2.22)a	19.04 (1.28)b	17.22 (1.76)c				
ALP (μmol h^−1^ g^−1^)	L10	4.18	1.04	2.81	2.68 (1.58)b	**	**	ns
	L14	6.30	1.58	4.11	4.00 (2.16)a			
	Mean	5.24 (1.54)a	1.31 (0.62)c	3.46 (0.97)b				
NPA (μmol h^−1^ g^−1^)	L10	13.91	6.68	8.85	9.81 (3.35)b	**	**	ns
	L14	19.33	7.19	10.74	12.42 (5.75)a			
	Mean	16.62 (3.83)a	6.93 (0.98)c	9.79 (1.39)b				
ACP (μmol h^−1^ g^−1^)	L10	124.65	58.96	122.73	102.11 (32.47)b	**	**	ns
	L14	138.89	74.01	134.44	115.78 (31.41)a			
	Mean	131.77 (8.33)a	66.48 (8.34)c	128.59 (6.55)b				
R6 growth stage								
URE (mg d^−1^ g^−1^)	L10	1.80	1.56	2.07	1.81 (0.23)b	**	**	*
	L14	2.11	1.85	2.56	2.17 (0.31)a			
	Mean	1.95 (0.18)b	1.71 (0.16)c	2.32 (0.27)a				
NAG (nM h^−1^ g^−1^)	L10	24.13	17.47	23.15	21.58 (3.21)b	**	**	ns
	L14	28.40	22.64	29.14	26.73 (3.18)a			
	Mean	26.26 (2.52)a	20.05 (2.88)b	26.15 (3.42)a				
LAP (nM h^−1^ g^−1^)	L10	32.73	27.90	25.65	28.76 (3.37)b	**	**	ns
	L14	35.29	30.49	27.52	31.10 (3.66)a			
	Mean	34.01 (2.29)a	29.19 (1.81)b	26.58 (1.39)c				
ALP (μmol h^−1^ g^−1^)	L10	8.61	1.68	6.90	5.73 (3.25)b	**	**	ns
	L14	11.45	2.12	9.36	7.64 (4.33)a			
	Mean	10.03 (1.76)a	1.90 (0.61)c	8.13 (1.84)b				
NPA (μmol h^−1^ g^−1^)	L10	26.26	8.38	10.07	14.90 (9.04)a	ns	**	ns
	L14	30.03	8.42	13.87	17.44 (9.96)a			
	Mean	28.15 (4.68)a	8.40 (1.45)b	11.97 (2.34)b				
ACP (μmol h^−1^ g^−1^)	L10	132.19	92.63	126.90	117.24 (18.88)b	**	**	ns
	L14	150.91	117.34	147.68	138.64 (16.25)a			
	Mean	141.55 (10.53)a	104.98 (13.57)b	137.29 (12.27)a				

Data are means (*n* = 3), values in parentheses represent standard deviation of the mean (*n* = 3). Different lowercase letters indicate significant difference among treatments (*p* < 0.05). *p < 0.05; **p < 0.01.

At the R6 growth stage, URE, NAG, LAP, ALP, and ACP activities were significantly different among cultivars and cropping systems, and URE activity showed a significant cultivars × cropping systems interaction. Continuous cropping induced significantly decreased URE, NAG, LAP, ALP, NPA, and ACP activities, and fertilization increased URE, NAG, LAP, ALP, and ACP activities under continuous cropping. L14 showed significantly higher URE, NAG, LAP, ALP, and ACP activities than L10 under CC treatment (Table 2).

### 3.3. Effects of continuous cropping on the copy numbers of nitrogen and phosphorus functional genes in soil of soybean cultivars with different tolerances

At the R1 growth stage, continuous cropping induced significantly decreased copy numbers of *AOA*, *AOB*, *nirK*, *nirK*, *phoA*, and *phoB* in soybean soil, and fertilization increased copy numbers of *AOA*, *AOB*, *nirK*, *nirK*, *phoA*, and *phoB* in soybean soil under continuous cropping. L14 had significantly higher copy numbers of *AOA*, *AOB*, *nirK*, *nirK*, *phoA*, and *phoB* than L10 under CC treatment. The copy numbers of soil nitrogen and phosphorus functional genes were in consistent trends between R1 and R6 growth stage, but the R6 growth stage showed higher copy numbers of soil nitrogen and phosphorus functional genes than the R1 growth stage (Table 3).

**Table 3:** Effects of crop rotation and continuous cropping on copy numbers of soil nitrogen and phosphorus functional genes.

Functional genes	Cultivar	CR	CC	CF
R1 growth stage				
*AOA* (10^7^ copies g^−1^ dry soil)	L10	1.72 ± 0.10bAB	0.98 ± 0.16dE	1.39 ± 0.16cCD
	L14	1.95 ± 0.08aA	1.15 ± 0.09dDE	1.51 ± 0.03cBC
*AOB* (10^6^ copies g^−1^ dry soil)	L10	3.24 ± 0.17abA	2.52 ± 0.16cB	2.99 ± 0.11bA
	L14	3.32 ± 0.18aA	2.63 ± 0.10cB	3.17 ± 0.09abA
*nirK* (10^5^ copies g^−1^ dry soil)	L10	8.70 ± 0.14bB	7.46 ± 0.10eD	8.34 ± 0.09cC
	L14	9.03 ± 0.12aA	7.72 ± 0.15dD	8.53 ± 0.12bcBC
*nifH* (10^6^ copies g^−1^ dry soil)	L10	2.43 ± 0.10bA	1.50 ± 0.07dC	1.29 ± 0.06eD
	L14	2.59 ± 0.06aA	1.68 ± 0.07cB	1.42 ± 0.04dCD
*phoA* (10^9^ copies g^−1^ dry soil)	L10	0.23 ± 0.01bAB	0.10 ± 0.01eE	0.17 ± 0.01dCD
	L14	0.27 ± 0.03aA	0.14 ± 0.01dDE	0.20 ± 0.01cBC
*phoB* (10^9^ copies g^−1^ dry soil)	L10	0.82 ± 0.01aAB	0.63 ± 0.03dC	0.72 ± 0.06bcBC
	L14	0.86 ± 0.04aA	0.67 ± 0.05cdC	0.79 ± 0.03abAB
R6 growth stage				
*AOA* (10^7^ copies g^−1^ dry soil)	L10	1.96 ± 0.17bAB	1.19 ± 0.15dD	1.44 ± 0.10cdCD
	L14	2.24 ± 0.20aA	1.29 ± 0.16dCD	1.63 ± 0.11cBC
*AOB* (10^6^ copies g^−1^ dry soil)	L10	3.45 ± 0.17aA	2.67 ± 0.18bB	3.32 ± 0.06aA
	L14	3.58 ± 0.06aA	2.72 ± 0.25bB	3.48 ± 0.31aA
*nirK* (10^5^ copies g^−1^ dry soil)	L10	9.41 ± 0.04bB	8.26 ± 0.20eD	9.05 ± 0.10cC
	L14	9.69 ± 0.05aA	8.49 ± 0.03dD	9.38 ± 0.12bB
*nifH* (10^6^ copies g^−1^ dry soil)	L10	2.55 ± 0.10bA	1.74 ± 0.10cdBC	1.43 ± 0.07eD
	L14	2.74 ± 0.11aA	1.85 ± 0.10cB	1.61 ± 0.06dCD
*phoA* (10^9^ copies g^−1^ dry soil)	L10	0.28 ± 0.02abAB	0.17 ± 0.02dC	0.23 ± 0.03cdBC
	L14	0.33 ± 0.04aA	0.20 ± 0.02cdC	0.25 ± 0.05bcABC
*phoB* (10^9^ copies g^−1^ dry soil)	L10	0.89 ± 0.06abA	0.74 ± 0.13bA	0.83 ± 0.12abA
	L14	0.97 ± 0.05aA	0.79 ± 0.08bA	0.88 ± 0.05abA

Lowercase letters represent significant difference (*p* < 0.05), uppercase letters represent extremely significant difference (*p* < 0.01).

### 3.4. Effects of continuous cropping on soil bacterial community structure of soybean cultivars with different tolerances

#### 3.4.1. Effects of continuous cropping on soil bacterial alpha diversity of soybean cultivars with different tolerances

The effects of CR and CC treatments on soil bacterial alpha diversity of soybean are shown in Supplementary Table S4. At the R1 growth stage, CC treatment decreased Observed_species index, Shannon index, Chao1 index and ACE index. L14 had higher Observed_species index, Shannon index, Chao1 index and ACE index than L10 under CC treatment. At the R6 growth stage, CC treatment increased Observed_species index, Chao1 index and ACE index, but decreased Shannon index. L14 had higher Observed_species index, Shannon index, Chao1 index and ACE index than L10 under CC treatment.

#### 3.4.2. Effects of continuous cropping on the number of soil bacteria at different taxonomic levels of soybean with different tolerances

At the R1 growth stage, the relative abundance of the top 10 bacterial phyla at the phylum level were *Proteobacteria*, *unidentified_Bacteria*, *Acidobacteriota*, *Actinobacteria*, *Bacteroidota*, *Gemmatimonadetes*, *Myxococcota*, *Chloroflexi*, *Verrucomicrobiota*, *Cyanobacteria*, which accounted for 86–89% of all bacterial sequences (Supplementary Figure S1). The relative abundance of each phylum was not significantly different between L14 and L10 under CC treatment.

At the R6 growth stage, the relative abundance of the top 10 bacterial phyla at the phylum level were *Proteobacteria*, *unidentified_Bacteria*, *Actinobacteria*, *Bacteroidota*, *Cyanobacteria*, *Acidobacteriota*, *Chloroflexi*, *Myxococcota*, *Verrucomicrobiota*, *Gemmatimonadetes*, which accounted for 87–89% of all bacterial sequences (Supplementary Figure S1). Under CC treatment, L14 had significantly higher relative abundance of *Proteobacteria*, *unidentified_Bacteria*, *Bacteroidota*, *Acidobacteriota*, and *Verrucomicrobiota* than L10 at the phylum level.

#### 3.4.3. Effects of continuous cropping on soil bacterial β diversity of different soybean cultivars

Principal co-ordinates analysis (PCoA) clearly showed that the first two components explained 12 and 7% of the total variability, respectively. The sample distance between R1 and R6 growth stage was relatively long, indicating that the bacterial community structure was significantly different between R1 and R6 growth stage. At the R1 growth stage, the distance of L14 and L10 on the first principal component axis (PC1) was significantly separated, indicating that cultivar was the main factor affecting the bacterial community structure. On the second principal component axis (PC2), the distance between CC and CR treatment was relatively great, indicating that continuous cropping was the second major factor affecting the bacterial community structure. At the R6 growth stage, the distance between cultivars and cropping systems was close, indicating that continuous cropping and cultivars had no significant effect on bacterial community structure at the R6 growth stage (Supplementary Figure S2).

#### 3.4.4. LEfSe analysis of soil bacterial communities of soybean cultivars with different tolerances under continuous cropping

**Figure 1:**
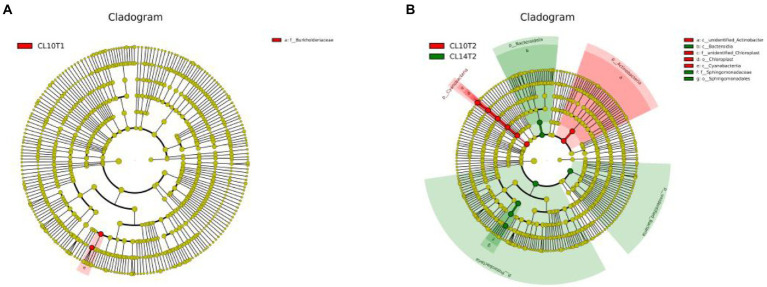
LDA Effect Size (LEfSe) analysis of soil bacteria communities of soybean cultivars under continuous cropping. **(A)** is the R1 growth stage, **(B)** is the R6 growth stage. CL10T1, at the R1 growth stage, L10 under CC treatment; CL10T2, at the R6 growth stage, L10 under CC treatment; CL14T2, at the R6 growth stage, L14 under CC treatment.

Under CC treatment, the difference analysis results among LEfSe species are shown in Figure 1. At the R1 growth stage, L10 had higher relative abundance of *Burkholderiaceae* than L14 at the family level (Figure 1A). At the R6 growth stage, L10 had higher relative abundance of *unidentified_Actinobacteria* and *Cyanobacteria* than L14 at the class level. L10 had higher relative abundance of *Chloroplast* than L14 at the order level. L10 had higher relative abundance of *unidentified_Chloroplast* than L14 at the family level. L14 had higher relative abundance of *Bacteroidia* than L10 at the class level. L14 had higher relative abundance of *Sphingomonadales* than L10 at the order level. L14 had higher relative abundance of *Sphingomonadaceae* than L10 at the family level (Figure 1B).

#### 3.4.5. Effects of soil properties on soil bacterial community structure of soybean cultivars with different tolerances

**Figure 2:**
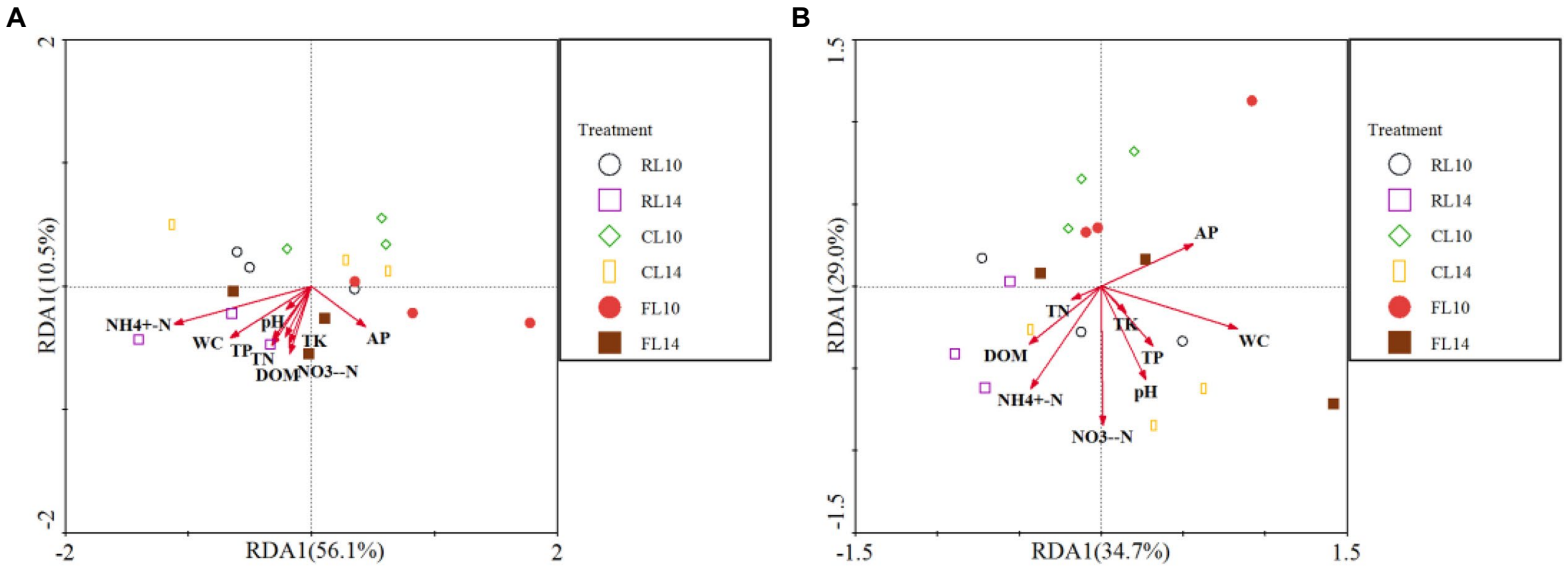
Redundancy analysis (RDA) of soil properties and bacterial community (phylum level). **(A)** is the R1 growth stage, **(B)** is the R6 growth stage. RL10, L10 under CR treatment; RL14, L14 under CR treatment; CL10, L10 under CC treatment; CL14, L14 under CC treatment; FL10, L10 under CF treatment; FL14, L14 under CF treatment.

The effect of soil properties on bacterial community structure was assessed by redundancy analysis (RDA) (Figure 2). At the R1 growth stage, for soybean soil bacterial community structure, the first two axes explained 67% of the variation of bacterial community structure. The values of NH_4_^+^-N (*F* = 8.93, *p* = 0.001) and NO_3_^−^-N (*F* = 5.70, *p* = 0.014) were positively correlated with the bacterial community structure. These results indicated that NH_4_^+^-N and NO_3_^−^-N value had a significant effect on the soil bacterial community structure (Figure 2A). At the R6 growth stage, for soybean soil bacterial community structure, the first two axes explained 64% of the variation of bacterial community structure. The value of AP (*F* = 3.10, *p* = 0.044) was positively correlated with the bacterial community structure. These results indicated that AP had a significant effect on the soil bacterial community structure (Figure 2B).

### 3.5. Effects of continuous cropping on soil bacterial community function of soybean cultivars with different tolerances

#### 3.5.1. Functional composition of soil bacterial community of soybean cultivars with different tolerances

**Figure 3:**
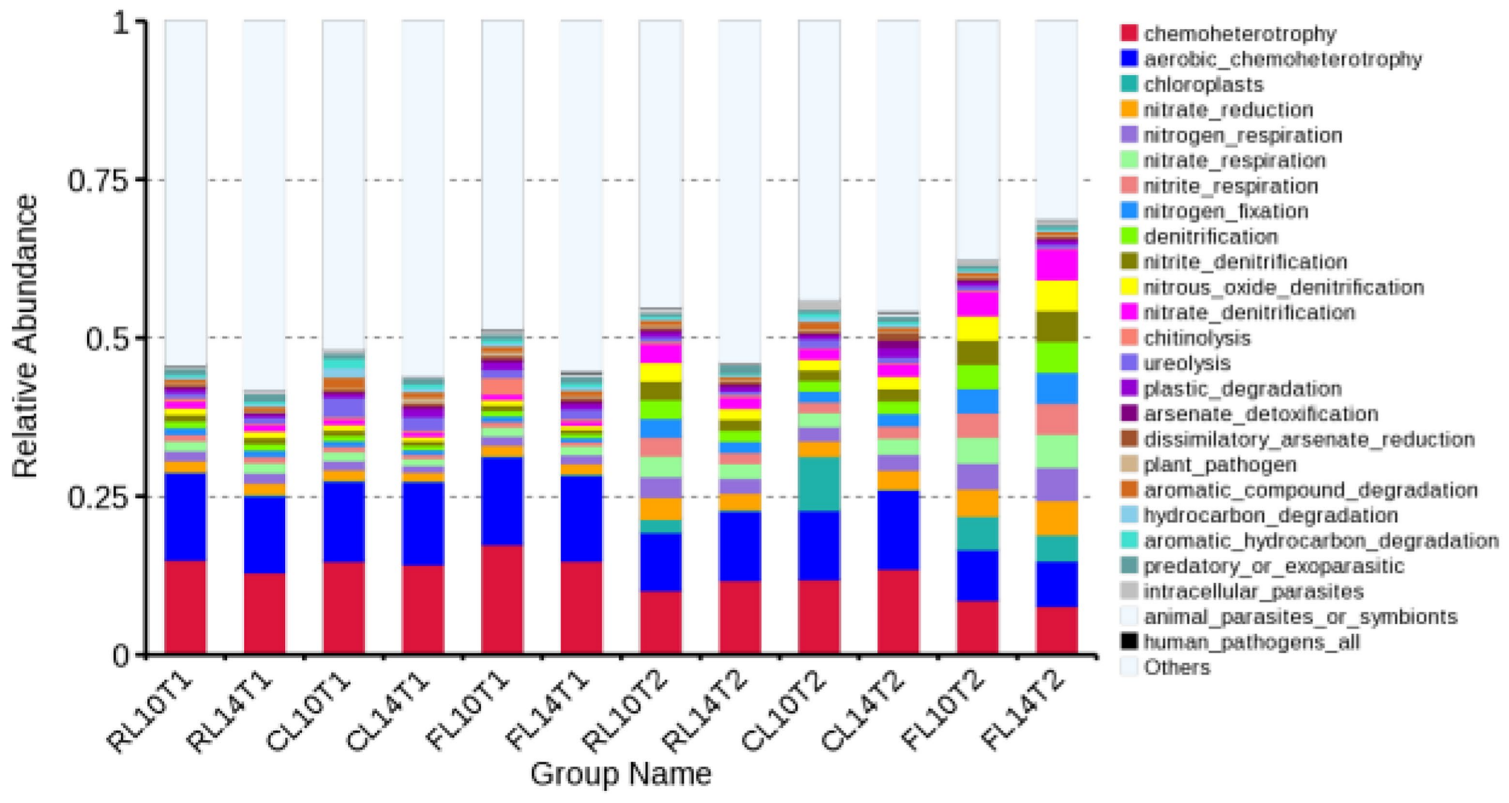
Relative abundance of functional bacteria in soil of soybean cultivars under crop rotation and continuous cropping. RL10T1, at the R1 growth stage, L10 under CR treatment; RL14T1, at the R1 growth stage, L14 under CR treatment; CL14T1, at the R1 growth stage, L14 under CC treatment; FL10T1, at the R1 growth stage, L10 under CF treatment; FL14T1, at the R1 growth stage, L14 under CF treatment; RL10T2, at the R6 growth stage, L10 under CR treatment; RL14T2, at the R6 growth stage, L14 under CR treatment; FL10T2, at the R6 growth stage, L10 under CF treatment; FL14T2, at the R6 growth stage, L14 under CF treatment.

At the R1 growth stage in the soybean soil bacterial community under CR and CC treatments, the relative abundance of the top 10 functional groups was chemoheterotrophy, aerobic chemoheterotrophy, chloroplasts, nitrate reduction, nitrogen respiration, nitrate respiration, nitrite respiration, nitrogen fixation, denitrification, nitrite denitrification, which accounted for 34–39% of the total bacterial functional abundance (Figure 3). L14 showed higher relative abundance of aerobic chemoheterotrophy than L10 under CC treatment. The relative abundance of the top 10 functional groups was consistent between R1 and R6 growth stage, which accounted for 37–54% of the total bacterial functional abundance (Figure 3). L14 had higher relative abundance of chemoheterotrophy and aerobic chemoheterotrophy than L10 under CC treatment.

#### 3.5.2. Effects of continuous cropping on functional β diversity of soil bacteria of soybean cultivars with different tolerances

**Figure 4:**
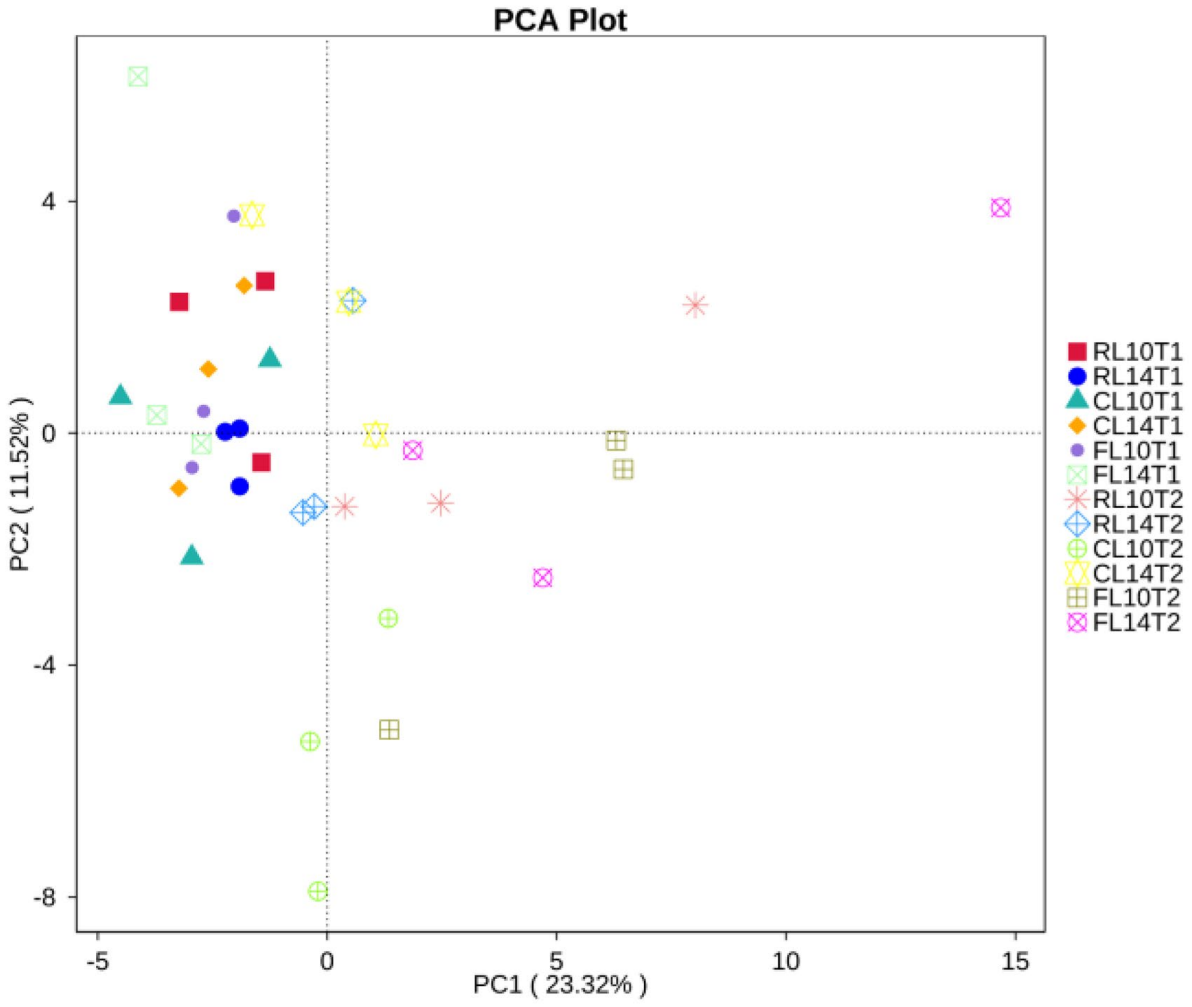
Principal component analyses (PCA) of soil functional community of soybean cultivars under crop rotation and continuous cropping.

Principal component analyses (PCA) clearly showed that the first two components explained 23 and 12% of the total variability, respectively. The sample distance between R1 and R6 growth stage was relatively long, indicating that the bacterial functional structure was significantly different between R1 and R6 growth stage. At the R1 growth stage, the distances between cultivars and cropping systems were close, indicating that and cultivars did not significantly effect of the bacteria functional structure at the R1 growth stage. At the R6 growth stage, the projections of CC and CR treatments on the first principal component axis (PC1) were significantly separated, indicating that continuous cropping was the main factor affecting the bacterial functional structure. On the second principal component axis (PC2), the distance between L14 and L10 was relatively great, especially under CC treatment, indicating that cultivar was the second major factor affecting the bacterial functional structure, especially under CC treatment (Figure 4).

#### 3.5.3. Effects of soil properties on soil bacterial functional structure of soybean cultivars with different tolerances

**Figure 5:**
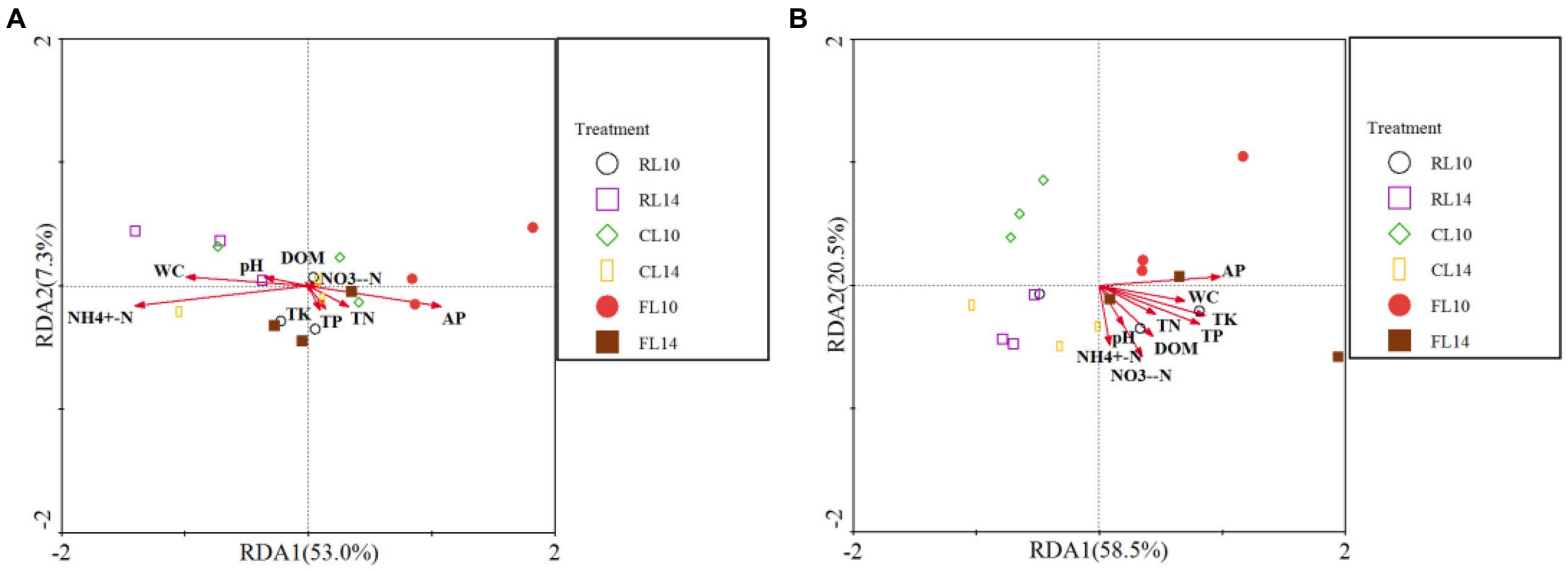
Redundancy analysis (RDA) of soil properties and bacterial functions. **(A)** is the R1 growth stage, **(B)** is the R6 growth stage.

The effect of soil properties on bacterial functional structure was assessed by RDA (Figure 5). At the R1 growth stage, for soybean soil bacterial functional structure, the first two axes explained 60% of the variation of bacterial functional structure. The value of NH_4_^+^-N (*F* = 4.82, *p* = 0.012) and NO_3_^−^-N (*F* = 4.59, *p* = 0.028) were positively correlated with the bacterial functional structure. These results indicated that NH_4_^+^-N and NO_3_^−^-N value had a significant effect on the soil bacterial functional structure (Figure 5A). At the R6 growth stage, the first two axes explained 79% of the variation of bacterial functional structure. The value of AP (*F* = 4.98, *p* = 0.009) and NO_3_^−^-N (*F* = 3.46, p = 0.028) were positively correlated with the bacterial functional structure. These results indicated that AP and NO_3_^−^-N had significant effects on the soil bacterial functional structure (Figure 5B).

#### 3.5.4. Co-occurrence network analysis of soil bacterial communities of soybean cultivars with different tolerances

**Figure 6:**
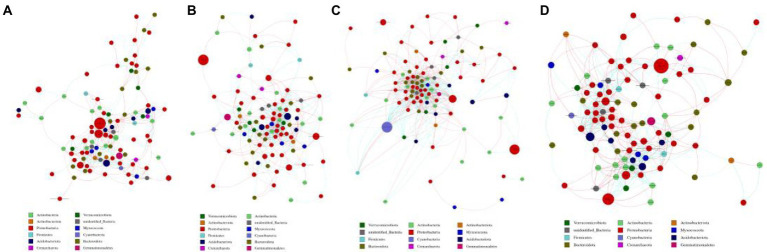
Co-occurrence network analysis of bacterial community in soybean soil. **(A)** is L10 at the R1 growth stage, **(B)** is L14 at the R1 growth stage, **(C)** is L10 at the R6 growth stage, **(D)** is L14 at the R6 growth stage. Different nodes represent different genera, node size represents the degree of connection of the genus, and the same color represents the same phylum level. The thickness of the connection between nodes is positively correlated with the absolute value of correlation coefficient of species interaction. The size of the node is proportional to the relative abundance of the phylum. Connections indicate significant correlation (Screening conditions: Spearman’s *ρ* > 0.6, *p* < 0.05).

The co-occurrence networks of soil bacterial community in L14 and L10 were constructed using Spearman coefficients among OTUs (Figure 6). At the R1 growth stage, L14 had a significantly higher number of nodes and edges of soil bacterial community than L10. The connectivity among soil bacterial communities in L10 was lower than L14, indicating the network associated with L10 was simpler. And the average degree of soil bacterial community in L10 was also lower than that in L14 (Figures 6A,B). The co-occurrence network of soil bacterial communities showed consistent trends between the R1 and R6 growth stages (Figures 6C,D).

## 4. Discussion

It was found that continuous cropping caused unbalanced changes in soil nutrients, which seriously affected the available nutrient content of soil and reduced soil productivity (Muhammad et al., 2020). The results of this study also showed that continuous cropping significantly decreased the contents of NH_4_^+^-N, NO_3_^−^-N, DOM, TK, TP, TN, and WC in soybean soil, and also significantly decreased soybean biomass and yield. The soil NH_4_^+^-N, NO_3_^−^-N, AP, DOM, TK, TP, TN, WC content, and pH value of L14 were higher than those of L10. The biomass and yield of L14 were higher than those of L10, while the yield reduction rate of L14 was lower than that of L10. The biochemical processes in soil are controlled by the activities of soil enzymes, which were mainly derived from the activities of soil microorganisms, and were an important biological index to characterize the vigorous degree of soil material, energy metabolism and soil quality level (Frankenberger and Dick, 1983; Pagliai and De Nobili, 1993). Different cropping systems would cause changes in soil pH, AP and other active components, leading to changes in soil microbial activity, and thus affecting soil nutrient cycling (Ehrenfeld et al., 1997). Soil nitrogen and phosphorus cycling enzymes (URE, NAG, LAP, ALP, NPA, and ACP) play an important role in soil nitrogen and phosphorus cycling. URE can convert organic nitrogen into inorganic nitrogen in soil; NAG can catalyze the terminal reaction of chitin degradation; LAP can hydrolyze leucine and other hydrophobic amino acids from the N-terminus of polypeptides; ALP, NPA and ACP can hydrolyze phosphate esters and they are usually associated with microbial metabolism (Schimel and Weintraub, 2003; Moorhead and Sinsabaugh, 2006; Wang et al., 2020). Different genotypes had significantly different effects on soil nitrogen and phosphorus cycling enzyme activities, which could affect nitrogen and phosphorus cycling (Márton et al., 2015). The results of this study showed that continuous cropping significantly decreased nitrogen and phosphorus cycling enzyme (URE, NAG, LAP, ALP, NPA, and ACP) activities and copy numbers of soil nitrogen and phosphorus cycling functional genes (*AOA*, *AOB*, *nirK*, *nirK*, *phoA*, *phoB*). The nitrogen and phosphorus enzyme activities and copy numbers of nitrogen and phosphorus functional genes of L14 were higher than those of L10. These results indicated that the continuous-cropping-tolerant soybean cultivar was more beneficial to the soil nitrogen and phosphorus cycles, improved soil nutrient utilization and nutrient supply capacity, and improved soybean plant material production capacity.

The different genotypes had significantly different effects on soil microorganisms, and the different genotypes would also affect soil microbial diversity and community structure (Huang et al., 2022; Xiao et al., 2022). In this study, it was found that root microorganisms were significantly different among different soybean cultivars. L14 showed higher Observed_species index, Shannon index, Chao1 index and ACE index than L10 under continuous cropping. These results indicated that the diversity of the soil bacterial community of the continuous-cropping-tolerant soybean cultivar was higher than that of the continuous-cropping-sensitive soybean cultivar under continuous cropping. This may be related to the differences in rhizosphere exudate composition and microbial activity habits among different cultivars (Liu et al., 2013).

Plant species diversity is a strong driver of soil microbial community structure (Fox et al., 2020). Different species of plants also affect soil microenvironment and organic matter input, thus changing soil biochemical activities and microbial community structure (Gsewell and Freeman, 2003; Zuber and Villamil, 2016). Different kinds and contents of organic matter secreted and released by roots of different cultivars change the activity and ecological niche of soil microorganisms (Lauren et al., 2021; Cui et al., 2022). In this study, PCoA showed that the projections of L14 and L10 on the principal component axis were significantly separated, indicating that cultivar was the main factor affecting the structure of soil bacterial community structure, and different cultivars had significant differences in soil bacterial community structure. The two cultivars had different responses to continuous cropping, which resulted in differences in the composition of root exudates and recruited different rhizosphere soil microorganisms (Kishore et al., 2013; Vicente et al., 2020). Based on the RDA of soil bacterial community and soil properties, it was found that NH_4_^+^-N, NO_3_^−^-N and AP had the most significant effects on soil bacterial community structure, indicating that the formation of different soil bacterial community structure was closely related to soil nitrogen and phosphorus sources under continuous cropping.

Microbial functional diversity is the ability of microorganisms to utilize a variety of substrates and biological processes, and it is an important mechanism for the soil microbial community to respond to soil environmental changes (Maier et al., 2009). In this study, FAPROTAX was used to predict the soil bacterial functional structure. It was found that chemoheterotrophy and aerobic chemoheterotrophy groups of different soybean cultivars were the main predictors of the soil bacterial functional structure under different cropping systems, and they were the dominant functional groups in soil (Figure 3). There was no input of other organic matter in continuous cropping soil, and the utilization of exogenous carbon was limited by nitrogen. Therefore, microorganisms need to strengthen the function of nitrogen acquisition to meet their demand for nitrogen (Kuzyakov, 2010). The soil bacterial functional groups were closely related to aboveground plants, so the different crop cultivars will cause the changes in soil bacterial community functional groups (Schlatter et al., 2015). This study showed that the projections of L14 and L10 on the principal component axis were significantly separated under continuous cropping, indicating that cultivar was the main factor affecting the soil bacterial functional structure. Based on the RDA of soil bacterial function and soil properties, it was found that NH_4_^+^-N, NO_3_^−^-N and AP had the most significant effects on soil bacterial functional structure, indicating that the differences of soil bacterial functional structure among soybean cultivars were closely related to soil nitrogen and phosphorus sources.

Microorganisms in the phyla *Proteobacteria* and *Acidobacteria* usually accounted for most of the soil bacterial communities (Fierer et al., 2007; Rao et al., 2021). Among the soil bacteria, *Proteobacteria*, *Acidobacteriota*, *Actinobacteria*, *Bacteroidota*, *Gemmatimonadetes* were the dominant phyla, and L14 showed significantly higher relative abundance of soil beneficial bacteria (*Proteobacteria*, *Bacteroidota*, *Acidobacteriota*, *Verrucomicrobiota*) than L10. *Proteobacteria* can adapt to a variety of plant rhizosphere microenvironments (Fierer et al., 2007; Peiffer et al., 2013), most of which have fix nitrogen and promote soil nitrogen cycle (Delmont et al., 2018); *Bacteroidota* play an important role in organic matter decomposition and polysaccharide metabolism (Larsbrink et al., 2017a,b; Huang et al., 2020; McKee et al., 2021); *Acidobacteriota* can degrade complex lignin and cellulose to provide sufficient energy and nutrients for soil microorganisms (Lynd et al., 2002; Pankratov et al., 2011; López-Mondéjar et al., 2015); *Verrucomicrobiota* can degrade cellulose and has the potential to synthesize antibiotics (Crits-Christoph et al., 2018). These results indicated that the continuous-cropping-tolerant soybean cultivar could recruit more beneficial bacteria and change the variability of soil bacterial community under continuous cropping, which was the main reason for the improvement of soil productivity.

Examination of the microbial co-occurrence network revealed the complex interactions among microorganisms, which reflect ecological linkages and processes (Barberán et al., 2012; Faust and Raes, 2012; Ramirez et al., 2018), and there is a strong positive correlation between crop yield and the diversity of key populations in the ecological network (Fan et al., 2021). The co-occurrence network of L14 had more nodes and edges than that of L10, indicating that the continuous-cropping-tolerant soybean cultivar had more bacterial interactions, larger network scale, and more complex and stable network structure (Liu et al., 2020). The increase of network complexity among microorganisms means the increase of functional connections in the network, and the increase of network complexity improved the efficiency of energy flow and material cycling in the ecosystem (Vries and Wallenstein, 2017). In addition, most of the nodes of L14 co-occurrence network had positive interactions, indicating that soil bacterial community of the continuous-cropping-tolerant soybean cultivar promoted each other and collaborative symbiosis, which made the whole soil bacterial ecosystem develop harmoniously.

## 5. Conclusion

In this study, the soil properties, rhizosphere soil microbial diversity, rhizosphere soil bacterial community structure and function among different soybean cultivars with continuous cropping tolerance were compared under continuous cropping. The results showed that biomass, soil nutrient content, soil nitrogen and phosphorus cycling enzyme activities, copy numbers of nitrogen and phosphorus functional genes, soil bacterial community diversity, soil bacterial community co-occurrence network complexity, and relative abundance of soil beneficial bacteria of L14 were significantly higher than those of L10, and the yield reduction rate of L14 was lower than that of L10 under continuous cropping. Soybean cultivars were the main factors affecting the structure and function of soil bacterial community under continuous cropping. Above all, the continuous-cropping-tolerant soybean cultivar recruited more beneficial bacteria, changed the structure and function of the microbial community, improved soil nitrogen and phosphorus cycling, and thus improved the productivity of continuous cropping soil to obtain higher yield.

## Data availability statement

The datasets generated of 16S rDNA sequencing for this study can be found in online repositories. The names of the repository/repositories and accession number(s) can be found at: https://www.ncbi.nlm.nih.gov/bioproject/PRJNA892778.

## Author contributions

WL, XY, and FX: conceived and designed the experiments. WL, NW, DH, and HS: performed experiments. WL, XY, XA, HW, HZ, SM, FX, and JW: wrote and revised the manuscript. All authors read and approved the final manuscript. All authors have read and agreed to the published version of the manuscript.

## Funding

This work was supported by the Project of Scientific Research in Education Department of Liaoning Province, China (Nos. LJKZ0630 and LJKZ0678) and the Opening Project of Key Laboratory of Soybean Biology of Chinese Education Ministry (SBKF12).

## Conflict of interest

The authors declare that the research was conducted in the absence of any commercial or financial relationships that could be construed as a potential conflict of interest.

## Publisher’s note

All claims expressed in this article are solely those of the authors and do not necessarily represent those of their affiliated organizations, or those of the publisher, the editors and the reviewers. Any product that may be evaluated in this article, or claim that may be made by its manufacturer, is not guaranteed or endorsed by the publisher.

## Footnotes

^1^ http://www.metware.cn

^2^ http://ccb.jhu.edu/software/FLASH/

^3^ http://qiime.org/scripts/split_libraries_fastq.html

^4^ https://github.com/torognes/vsearch/
