# Supplementary material for: Continuous-cropping-tolerant soybean cultivars alleviate continuous cropping obstacles by improving structure and function of rhizosphere microorganisms
Source: Front Microbiol. 2023 Jan 4;13:1048747. doi: 10.3389/fmicb.2022.1048747 (PMC9846356; doi:10.3389/fmicb.2022.1048747)
Supplement: Supplementary file 1 [file Data_Sheet_1.docx]

Supplementary Material

# Supplementary Figures


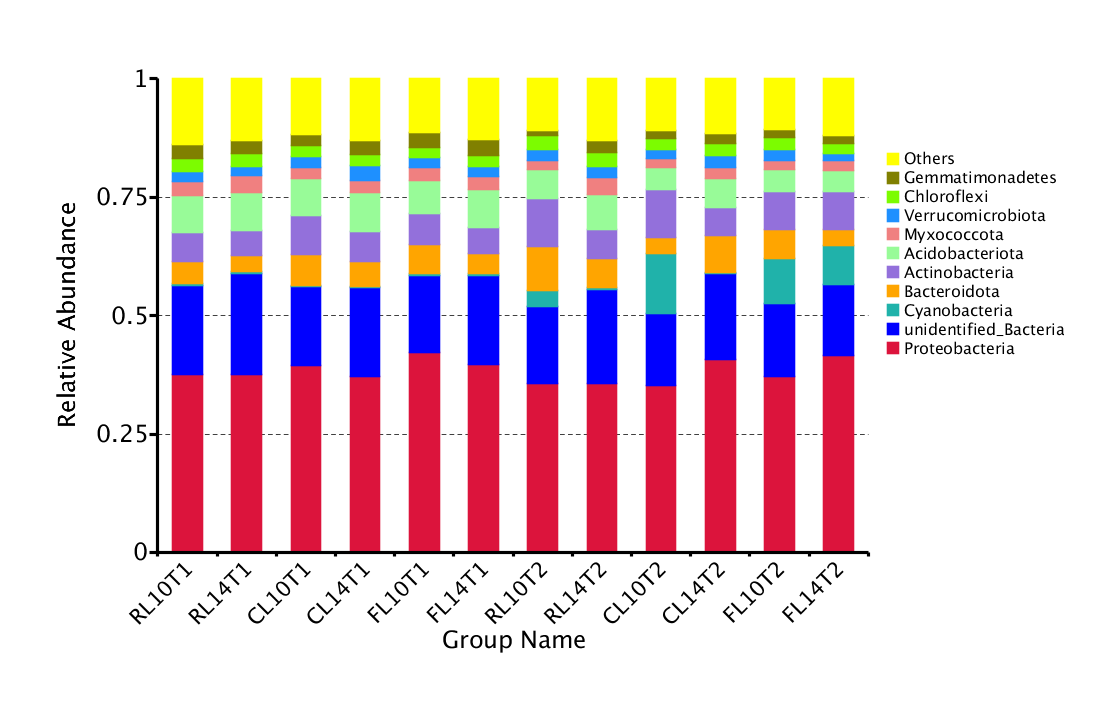


**Figure S1.** Phylum level relative abundance of bacteria in soils of soybean cultivars in crop rotation and continuous cropping.


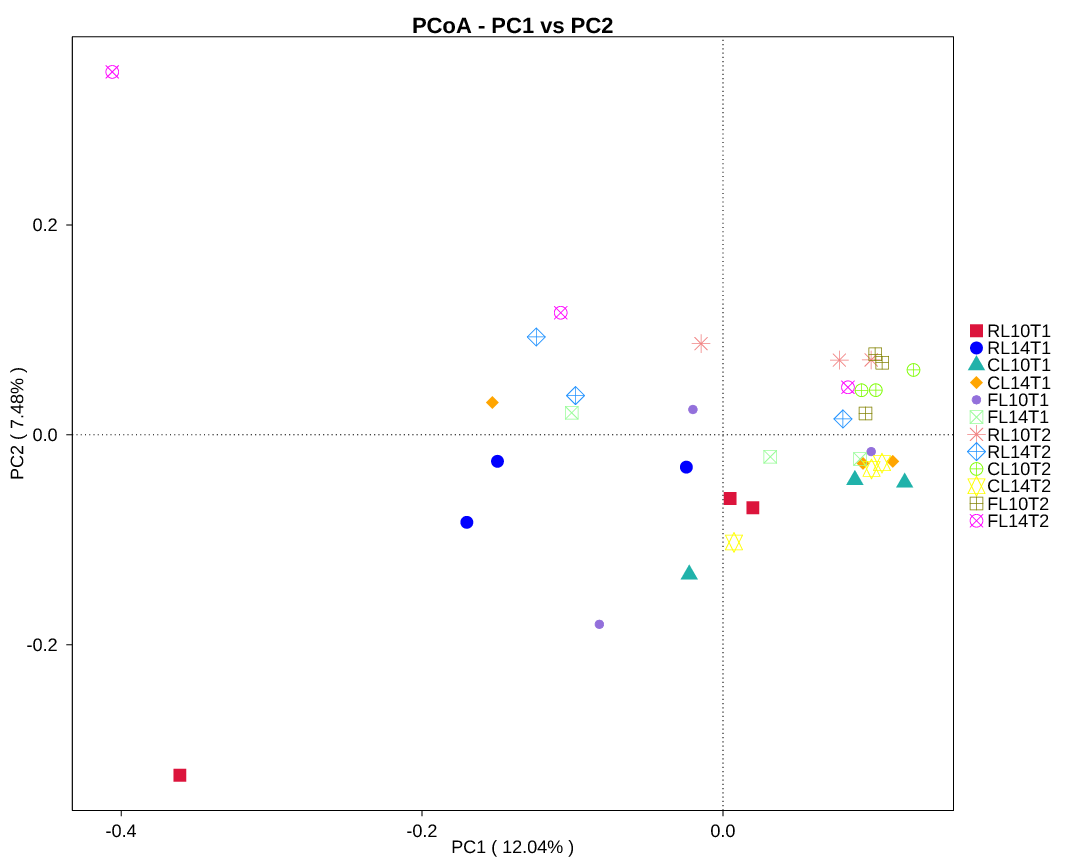


**Figure S2.** Principal co-ordinates analysis (PCoA) of soil bacterial communities of soybean cultivars under crop rotation and continuous cropping.

# Supplementary Tables

**Table S1.** Primers used for quantitative PCR.

| Functional genes | Primer sequence |
| --- | --- |
| *phoA* | Forward CAGTAGGAGCCATGAGCGAG |
|  | Reverse CCGGTTGTATGGTCAGCAGT |
| *phoB* | Forward GCGCCATGAGTGAAATGGAC |
|  | Reverse CGTTGCGACAACCAATGTGT |
| *AOA* | Forward S(C/G)TAATGGTCTGGCTTAGACG |
|  | Reverse GCGGCCATCCATCTGTATGT |
| *AOB* | Forward GGGGTTTCTACTGGTGGT |
|  | Reverse CCCCTCKGSAAAGCCTTCTTC |
| *nirK* | Forward GG(A/C)ATGGTK(G/T)CC(C/G)TGGCA |
|  | Reverse GCCTCGATCAG(A/G)TT(A/G)TGG |
| *nifH* | Forward AAAGG(C/T)GG(A/T)ATCGG(C/T)AA(A/G)TCCACCAC |
|  | Reverse TTGTT(G/C)GC(G/C)GCRTACAT(G/C)GCCATCAT |

**Table S2.** Effects of crop rotation and continuous cropping treatments on soybean soil properties at the R1 growth stage.

| Soil properties | Cultivar | CR | CC | CF | Mean | Cultivar | Cropping system | Cultivar×Cropping system |
| --- | --- | --- | --- | --- | --- | --- | --- | --- |
| NH_4_^+^-N (mg kg^-1^) | L10 | 8.45 | 5.53 | 6.94 | 6.97(1.54)b | ** | ** | ns |
|  | L14 | 11.30 | 8.91 | 10.05 | 10.09(1.64)a |  |  |  |
|  | Mean | 9.87(1.78)a | 7.22(2.15)b | 8.49(2.20)ab |  |  |  |  |
| NO_3_^-^-N (mg kg^-1^) | L10 | 1.02 | 0.58 | 1.45 | 1.02(0.39)b | ** | ** | * |
|  | L14 | 1.68 | 1.13 | 1.63 | 1.48(0.29)a |  |  |  |
|  | Mean | 1.35(0.38)b | 0.86(0.32)c | 1.54(0.14)a |  |  |  |  |
| AP (mg kg^-1^) | L10 | 8.88 | 7.37 | 28.72 | 14.99(10.35)b | ** | ** | * |
|  | L14 | 10.64 | 11.12 | 30.30 | 17.35(9.73)a |  |  |  |
|  | Mean | 9.76(1.13)b | 9.25(2.12)b | 29.51(1.14)a |  |  |  |  |
| DOM (mg kg^-1^) | L10 | 88.81 | 53.78 | 86.23 | 76.27(17.76)b | * | ** | ns |
|  | L14 | 96.39 | 65.13 | 99.01 | 86.84(18.03)a |  |  |  |
|  | Mean | 92.60(9.79)a | 59.46(8.14)b | 92.62(9.15)a |  |  |  |  |
| TK (g kg^-1^) | L10 | 17.61 | 15.65 | 19.72 | 17.66(1.78)b | ** | ** | ns |
|  | L14 | 19.72 | 18.53 | 22.81 | 20.36(1.92)a |  |  |  |
|  | Mean | 18.66(1.20)b | 17.09(1.58)c | 21.26(1.70)a |  |  |  |  |
| TP (g kg^-1^) | L10 | 2.22 | 1.29 | 3.34 | 2.29(0.98)b | * | ** | ns |
|  | L14 | 3.01 | 1.96 | 3.80 | 2.93(0.88)a |  |  |  |
|  | Mean | 2.62(0.66)b | 1.63(0.50)c | 3.57(0.44)a |  |  |  |  |
| TN (g kg^-1^) | L10 | 1.69 | 1.18 | 1.98 | 1.62(0.48)a | ns | ** | ns |
|  | L14 | 2.18 | 1.30 | 2.27 | 1.92(0.56)a |  |  |  |
|  | Mean | 1.94(0.59)a | 1.24(0.14)b | 2.13(0.22)a |  |  |  |  |
| pH | L10 | 5.72 | 5.87 | 5.98 | 5.86(0.12)b | ** | ** | ** |
|  | L14 | 6.07 | 6.14 | 6.07 | 6.10(0.05)a |  |  |  |
|  | Mean | 5.90(0.19)b | 6.00(0.16)a | 6.03(0.07)a |  |  |  |  |
| WC (%) | L10 | 12.56 | 11.55 | 10.50 | 11.54(1.33)b | ** | ** | ** |
|  | L14 | 15.60 | 10.34 | 14.43 | 13.46(2.64)a |  |  |  |
|  | Mean | 14.08(2.06)a | 10.95(1.21)b | 12.46(2.38)b |  |  |  |  |

Note:NH4^+^-N, ammonium nitrogen; NO3^-^-N, nitrate nitrogen; AP, available phosphorus; DOM, dissolved organic matter; TK, total potassium; TP, total phosphorus; TN, total nitrogen; WC, water content. Data are means (n = 3), values in parentheses represent standard deviation of the mean (n = 3). Different lowercase letters after values represent significant differences among all treatments based on Duncan’s test (P < 0.05). *P < 0.05; **P < 0.01.

**Table S3.** Effects of crop rotation and continuous cropping treatments on soybean soil properties at the R6 growth stage.

| Soil properties | Cultivar | CR | CC | CF | Mean | Cultivar | Cropping system | Cultivar×Cropping system |
| --- | --- | --- | --- | --- | --- | --- | --- | --- |
| NH_4_^+^-N (mg kg^-1^) | L10 | 16.37 | 11.65 | 15.25 | 14.42(2.49)b | ** | ** | ns |
|  | L14 | 22.54 | 16.57 | 21.50 | 20.20(3.25)a |  |  |  |
|  | Mean | 19.46(3.73)a | 14.11(3.00)b | 18.37(3.83)a |  |  |  |  |
| NO_3_^-^-N (mg kg^-1^) | L10 | 1.81 | 1.11 | 2.24 | 1.72(0.50)b | ** | ** | ns |
|  | L14 | 3.27 | 2.67 | 3.48 | 3.14(0.40)a |  |  |  |
|  | Mean | 2.54(0.81)b | 1.89(0.87)c | 2.86(0.69)a |  |  |  |  |
| AP (mg kg^-1^) | L10 | 17.50 | 16.96 | 37.10 | 23.85(9.97)b | ** | ** | ns |
|  | L14 | 22.25 | 20.24 | 41.11 | 27.87(10.08)a |  |  |  |
|  | Mean | 19.88(2.82)b | 18.60(1.91)b | 39.10(2.80)a |  |  |  |  |
| DOM (mg kg^-1^) | L10 | 139.86 | 102.36 | 141.48 | 127.90(20.63)b | ** | ** | ns |
|  | L14 | 152.02 | 116.19 | 154.98 | 141.06(19.35)a |  |  |  |
|  | Mean | 145.94(9.27)a | 109.27(9.24)b | 148.23(10.85)a |  |  |  |  |
| TK (g kg^-1^) | L10 | 20.91 | 17.69 | 22.69 | 20.43(2.21)b | ** | ** | * |
|  | L14 | 22.65 | 19.18 | 25.13 | 22.32(2.60)a |  |  |  |
|  | Mean | 21.78(0.96)b | 18.44(0.86)c | 23.91(1.39)a |  |  |  |  |
| TP (g kg^-1^) | L10 | 3.00 | 2.05 | 3.36 | 2.80(1.13)a | ns | * | ns |
|  | L14 | 3.43 | 2.44 | 4.26 | 3.38(0.10)a |  |  |  |
|  | Mean | 3.22(1.31)ab | 2.24(0.45)b | 3.81(0.73)a |  |  |  |  |
| TN (g kg^-1^) | L10 | 2.07 | 1.55 | 2.49 | 2.03(0.60)a | ns | * | ns |
|  | L14 | 2.57 | 1.74 | 2.73 | 2.34(0.62)a |  |  |  |
|  | Mean | 2.32(0.63)a | 1.64(0.33)b | 2.61(0.42)a |  |  |  |  |
| pH | L10 | 5.61 | 5.71 | 5.83 | 5.71(0.10)b | ** | ** | ns |
|  | L14 | 5.91 | 6.00 | 6.08 | 5.99(0.08)a |  |  |  |
|  | Mean | 5.76(0.17)c | 5.85(0.16)b | 5.95(0.14)a |  |  |  |  |
| WC (%) | L10 | 14.79 | 14.36 | 15.70 | 14.94(0.95)a | ns | ** | * |
|  | L14 | 13.37 | 16.15 | 16.40 | 15.31(1.65)a |  |  |  |
|  | Mean | 14.08(1.19)b | 15.26(1.21)a | 16.05(0.83)a |  |  |  |  |

Note:NH4^+^-N, ammonium nitrogen; NO3^-^-N, nitrate nitrogen; AP, available phosphorus; DOM, dissolved organic matter; TK, total potassium; TP, total phosphorus; TN, total nitrogen; WC, water content. Data are means (n = 3), values in parentheses represent standard deviation of the mean (n = 3). Different lowercase letters after values represent significant differences among all treatments based on Duncan’s test (P < 0.05). *P < 0.05; **P < 0.01.

**Table S4.** Effects of crop rotation and continuous cropping treatments on soil bacterial α diversity of soybean.

| Sample name | Observed_species | Shannon | Simpson | Chao1 | ACE |
| --- | --- | --- | --- | --- | --- |
| RL10T1 | 3085.00±484.24aA | 9.22±0.24aA | 0.993±0.001aA | 3316.03±323.29abA | 3398.98±359.50abA |
| RL14T1 | 3139.33±186.00aA | 9.34±0.13aA | 0.994±0.001aA | 3495.02±149.91aA | 3605.17±196.22aA |
| CL10T1 | 2716.33±134.69aA | 9.04±0.14aA | 0.993±0.001aA | 3042.51±138.56bA | 3089.92±150.73bA |
| CL14T1 | 2801.00±133.30aA | 9.15±0.03aA | 0.993±0.001aA | 3130.96±162.32abA | 3188.17±180.15abA |
| FL10T1 | 2801.33±218.22aA | 9.02±0.41aA | 0.990±0.006aA | 3154.13±267.87abA | 3230.87±279.81abA |
| FL14T1 | 2875.33±102.11aA | 9.21±0.04aA | 0.993±0.000aA | 3207.22±106.98abA | 3287.94±101.46abA |
| RL10T2 | 2520.00±11.73cB | 8.68±0.39aA | 0.988±0.008aA | 2848.20±84.50cB | 2907.96±103.02cB |
| RL14T2 | 2913.00±68.24abAB | 9.23±0.06aA | 0.994±0.001aA | 3247.04±51.06abAB | 3302.53±53.19abAB |
| CL10T2 | 2662.00±223.81bcAB | 8.46±0.49aA | 0.978±0.013aA | 2986.23±255.20bcAB | 3096.12±311.30bcAB |
| CL14T2 | 2906.33±179.72bcAB | 8.99±0.25aA | 0.990±0.004aA | 3269.88±206.66abAB | 3349.34±222.20abAB |
| FL10T2 | 2690.00±173.86bcAB | 8.44±0.64aA | 0.978±0.015aA | 3005.55±183.33bcAB | 3107.03±217.32bcAB |
| FL14T2 | 3069.33±119.06aA | 8.27±1.10aA | 0.955±0.051aA | 3433.45±163.29aA | 3593.26±204.99aA |

Note: RL10T1, at the R1 growth stage, L10 under CR treatment; RL14T1, at the R1 growth stage, L14 under CR treatment; CL10T1, at the R1 growth stage, L10 under CC treatment; CL14T1, at the R1 growth stage, L14 under CC treatment; FL10T1, at the R1 growth stage, L10 under CF treatment; FL14T1, at the R1 growth stage, L14 under CF treatment; RL10T2, at the R6 growth stage, L10 under CR treatment; RL14T2, at the R6 growth stage, L14 under CR treatment; CL10T2, at the R6 growth stage, L10 under CC treatment; CL14T2, at the R6 growth stage, L14 under CC treatment; FL10T2, at the R6 growth stage, L10 under CF treatment; FL14T2, at the R6 growth stage, L14 under CF treatment. Lowercase letters represent significant difference (P<0.05), uppercase letters represent extremely significant difference (P<0.01).
